# Percutaneous Vesselplasty for the Treatment of Vertebral Osteoporotic Collapse Fractures

**DOI:** 10.7759/cureus.68695

**Published:** 2024-09-05

**Authors:** Prachita Agrawal, Pankaj Banode, Vadlamudi Nagendra, Raj Joshi, Shubham Agrawal, Jubin John, Neha Nawale

**Affiliations:** 1 Interventional Radiology, Jawaharlal Nehru Medical College, Datta Meghe Institute of Higher Education and Research, Wardha, IND; 2 Interventional Radiology, Jawaharlal Nehru Medical College, Datta Meghe Institute of Higher Education and Research,Wardha, Wardha, IND; 3 Neurology, Sawai Man Singh Medical College, Jaipur, IND; 4 Radiodiagnosis, Jawaharlal Nehru Medical College, Datta Meghe Institute of Higher Education and Research, Wardha, IND; 5 Clinical Embryology, School of Allied Health Sciences, Datta Meghe Institute of Higher Education and Research, Wardha, IND

**Keywords:** fluoroscopic examination, polymethylmethacrylate (pmma), vertebral body, vertebral bone, vesselplasty

## Abstract

A 78-year-old woman with vertebral compression fractures (VCFs) and chronic back pain was the subject of this case study. To treat symptomatic VCFs, less invasive procedures such as balloon kyphoplasty and vertebroplasty were developed. Vertebroplasty involves injecting polymethylmethacrylate (PMMA) into the vertebral body to mechanically stabilize it. However, there was a risk that PMMA could leak, potentially causing a pulmonary embolism or neurological problems. A modified vertebroplasty called balloon kyphoplasty reduced the chance of leakage but was not without complications. It used a high-pressure balloon to create a cavity before cement injection. An alternative procedure, called vesselplasty, aimed to reduce bone filler leakage, minimize void formation, and restore vertebral height. When vesselplasty was introduced, bone cement was held in place by a polyethylene terephthalate (PET) balloon container that expanded inside the vertebral body to lower the risk of leakage. Vesselplasty was shown to be a more secure and successful treatment for VCFs, especially in osteoporotic patients with peripheral wall injury. Vesselplasty was a potentially effective treatment for osteoporotic VCF patients in correcting kyphosis, restoring vertebral height, and reducing pain. During a six-month follow-up period, post-operative examinations following vesselplasty demonstrated considerable pain reduction, improved vertebral height, and superior overall health outcomes.

## Introduction

Vesselplasty was proven to be a more stable and successful treatment for vertebral compression fractures (VCFs), particularly in patients with osteoporotic peripheral wall injuries. According to the study, patients with osteoporotic VCFs benefited from vesselplasty to reduce discomfort, correct kyphosis, and restore spinal height. During a six-month follow-up period after vesselplasty, postoperative tests showed considerable pain reduction, improved vertebral height, and better overall health outcomes [1]. Osteoporosis, which can be intrinsic (related to aging or postmenopausal) or secondary (induced by a range of disorders and medications), was the most common cause of VCFs. Another significant cause of VCFs was tumor infiltration associated with metastasis, similar to that of myeloma. The necessity for corticosteroids in cancer patients' treatment often results in secondary osteoporosis, which leads to the synthesis of additional VCFs. VCFs can cause kyphotic angulation of the spine, severe and prolonged pain, a decrease in forced vital capacity, and other comorbidities such as early satiety-induced weight loss and low psychological well-being [2]. The standard approach for treating symptomatic VFCs includes medical therapy, bed rest, analgesics, rehabilitation, and external bracing. Moreover, bed rest increased the risk of subsequent fractures by hastening the resorption of bone. Generally, spinal instability or neurologic deficits were the only conditions that necessitated surgery. However, surgical fixation frequently failed due to the poor quality of osteoporotic bone. For these reasons, doctors were investigating state-of-the-art techniques for pain management and functional restoration to help patients resume their everyday lives [3].

Two minimally invasive percutaneous treatments were developed to treat symptomatic: balloon kyphoplasty and vertebroplasty. The first case of percutaneous vertebroplasty was reported in 1984. In vertebroplasty, a polymethylmethacrylate (PMMA) percutaneous injection was administered into the fractured vertebral body as part of an imaging-guided procedure. The mechanical stability of the vertebral body seemed to be the most probable explanation for pain reduction after vertebroplasty, although this notion is still under investigation. In most ex vivo studies, cement injections were used to restore the stiffness and strength of the vertebral body. The main danger with vertebroplasty was PMMA leaking into veins, which could result in a pulmonary embolism, or into neural foramina or the spinal canal, potentially causing a nervous system disorder [4]. A modified method known as percutaneous balloon kyphoplasty involved inflating a high-pressure balloon (KyphX Inflatable Bone Tamps, Kyphon) inside the collapsed vertebral body. Next, a percutaneous injection of bone cement was made into the cavity created by a balloon. This procedure was created and implanted for the first time in 1998. Balloon kyphoplasty lowered the risk of cement extravasation because low-pressure cavities were filled with high-viscosity cement, and the compressed trabeculae created new bone boundaries [5].

A novel and exciting substitute for vertebroplasty and kyphoplasty is vesselplasty. The goals of vesselplasty are to prevent bone filler material from leakage, elevate the vertebral body to its original height, and reduce the void that develops in the vertebral body. A-Spine Holding Group Corporation chairman Jerry Lin created the vesselplasty treatment (Taipei, Taiwan). In 2004, Darwono utilized it for the first time (Darwono B, 2004 Triennial Asia Pacific Orthopedic Congress, Kuala Lumpur, Malaysia). Instead of using a balloon to create a cavity, vesselplasty used a polyethylene terephthalate (PET) balloon container (Vessel-X, A-Spine Holding Group Corporation) to restore the height of the vertebral body. This container held bone cement and functioned as a vertebral body expander. PMMA is injected into the spine to expand it once delivered in its reduced form. A small quantity of bone cement is interdigitated within the PET channel by passing through its porous fibers to strengthen the vertebral body. Since most of the cement is contained in the expanding artificial vessel, this technique theoretically resolves the issue of cement leakage from the vertebral body. Vesselplasty offered a safe method of handling VCFs [6]. This study assessed vesselplasty's safety and efficacy in treating symptomatic VCFs.

## Case presentation

Patient information

A 78-year-old female history patient had a history of persistent back pain that had spread to both flanks and lower limbs. She reported that the pain increased after a minor trauma and had persisted for four to five years.

Medical/surgical history

Investigation

**Figure 1:**
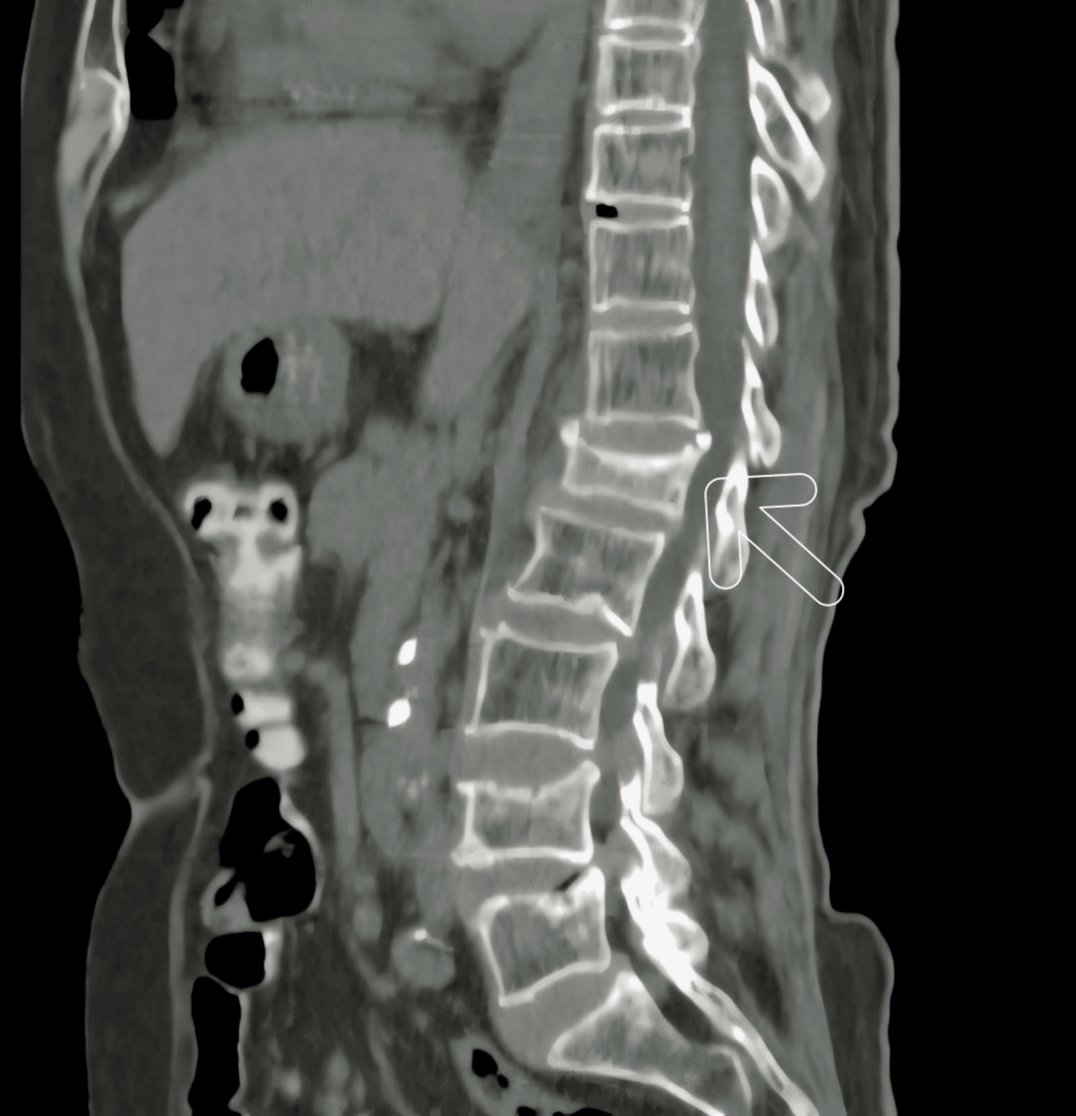
Computed tomography (CT) of the lumbar spine The arrow indicates the central wedge compression fracture of the L1 vertebral body and generalized osteopenia.

A lumbar spine computed tomography (CT) scan revealed a central wedge compression fracture of the L1 vertebral body with a fracture fragment encroaching on the spinal canal, along with widespread osteopenia. At the L4 spinal level, a central wedge compression fracture was also observed, as shown in Figure 1.

**Figure 2:**
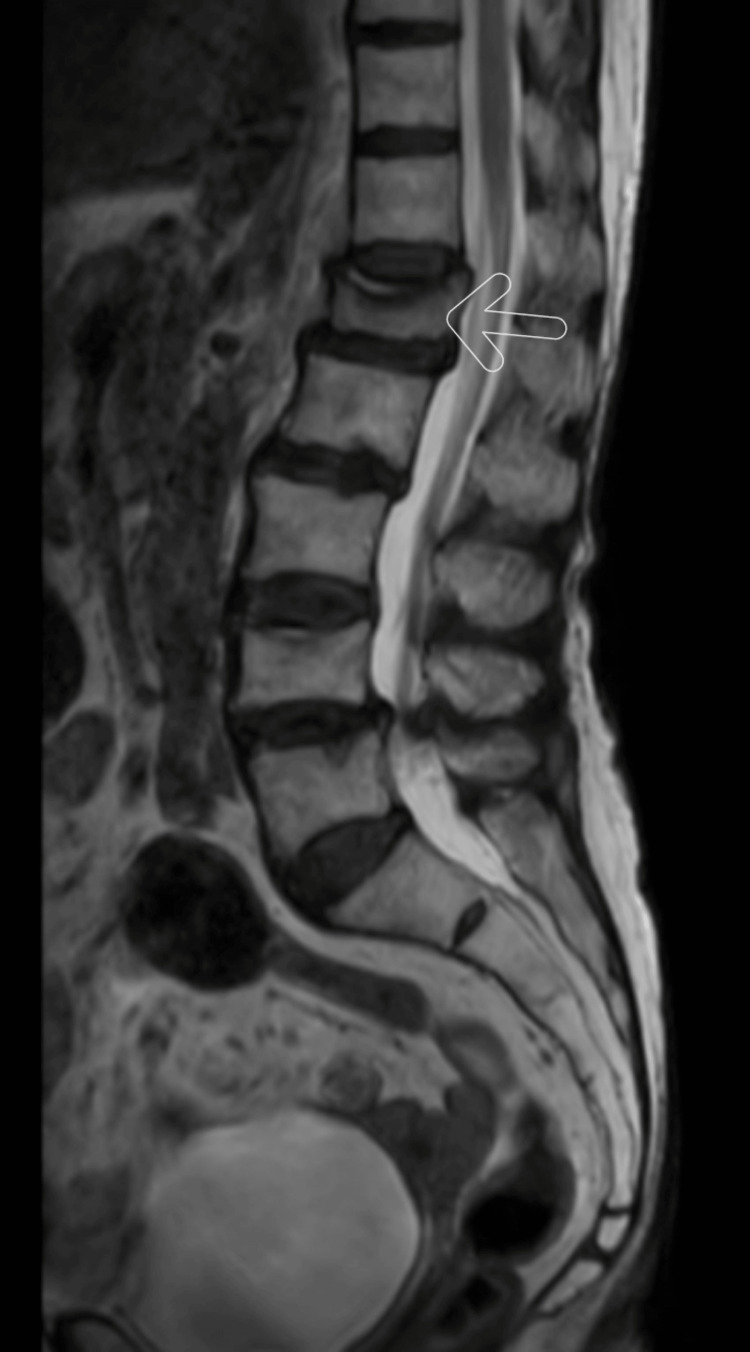
Sagittal MRI lumbar spine showing anterior wedge collapse of the L1 vertebra

**Figure 3:**
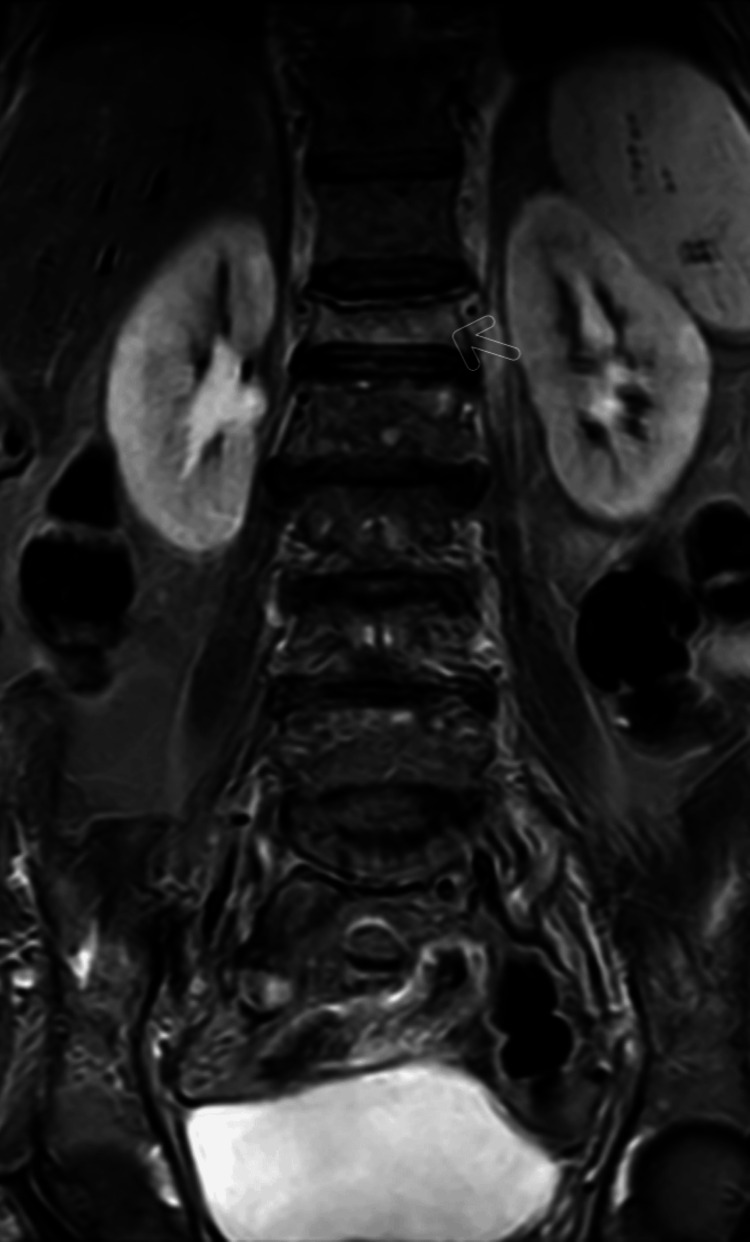
Coronal MRI lumbar spine showing wedge collapse fracture of L1

In addition, the central wedging of L4 and anterior wedge collapse of L1 were seen on the magnetic resonance imaging (MRI) of the lumbar spine. As shown in Figures 2 and Figure 3, osteophytes were marginal at several levels.

The patient underwent a comprehensive endocrine evaluation to rule out endocrinopathies; no abnormalities were found.

Treatment

**Figure 4:**
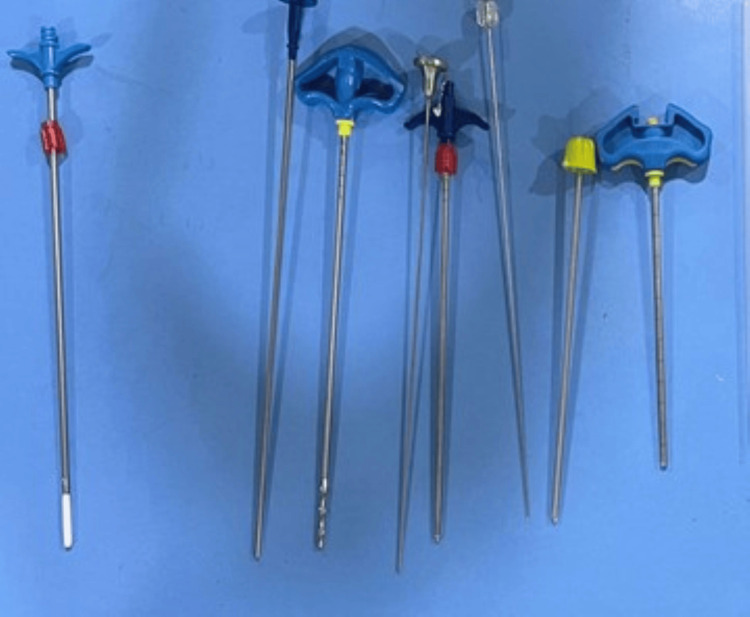
Instruments of vesselplasty From right to left: cannula tube, stylet, pushing rod and Vessel-X bone filling container, pushing rod, precision drill, pushing rod, and Vessel-X bone filling container

After the diagnosis was made, the course of treatment was discussed. Following our explanation of the advantages and disadvantages of several surgical techniques, the patient and her family decided on vesselplasty. The patient lay prone on an angiography table for the procedure. The instrument used for surgery is shown in Figure 4.

**Figure 5:**
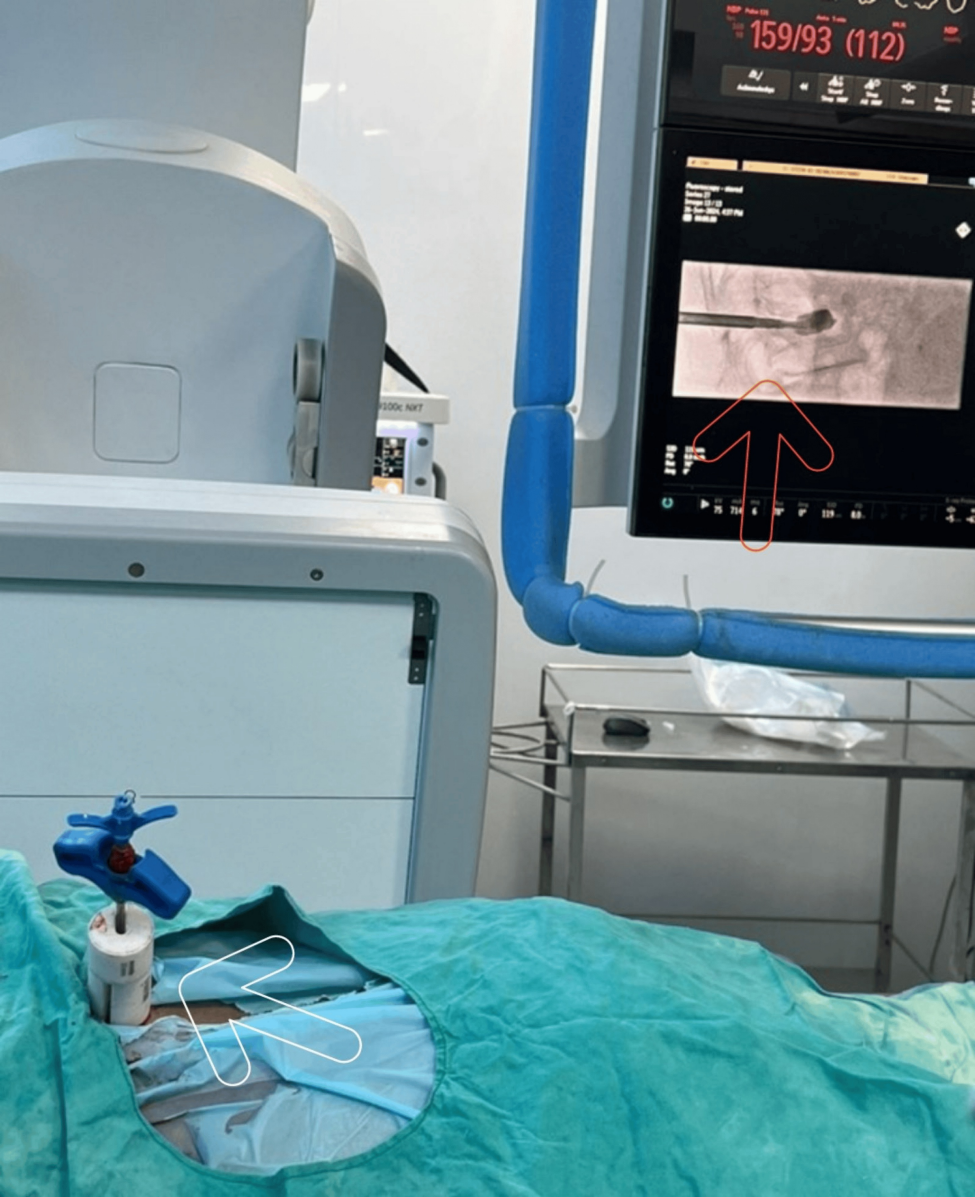
Peri-procedural images of vesselplasty The white arrow indicates bone cement being injected into the vertebral body of the patient with a vertebral compression fracture. The red arrow indicates the lateral fluoroscopy view demonstrating the injection of bone cement through the bone filling mesh container.

The collapsed vertebral body (L1 VB) was initially identified in the lateral and anteroposterior planes with the help of fluoroscopic guidance. To place the pedicle in the craniocaudal plane and the anteroposterior oblique plane for a lateral-to-medial approach, it was then isolated in the lateral plane. The patient had intravenous fentanyl and midazolam while under conscious anesthesia. Topical 0.25% bupivacaine was used as a local anesthetic on the pedicle. A tiny skin incision was created to construct a pathway through the pedicle, and a 10-gauge bone access needle with an inner stylet was introduced into the L1 vertebral body. Two or three millimeters in front of the point where the bone access needle was terminated was the posterior wall of the vertebral body. The balloon catheter needed a tunnel, which was made possible by advanced drill technology. The balloon was pushed through the needle to the anterior region of the VB after the drill trocar was removed. A C-arm machine was used to monitor the balloon's inflation process when it reached its maximum pressure of 200 psi. Under fluoroscopy guidance, PMMA cement was injected into the balloon as it started to get doughy. An average of two milliliters of cement injection was given. After that, the pushing rod and vessel introducer were taken out (Figure 5).

To reduce the local inflammatory response associated with the surgery, 1 mg/kg of IV methylprednisolone was administered after the procedure. The patient was required to lie down for six hours after the procedure to monitor vital signs, neurological function, and sensory-motor abilities in their lower limbs. After a 24-hour hospital stay, the patient was examined by a neurologist and interventional radiologist before being discharged.

Follow-up

**Figure 6:**
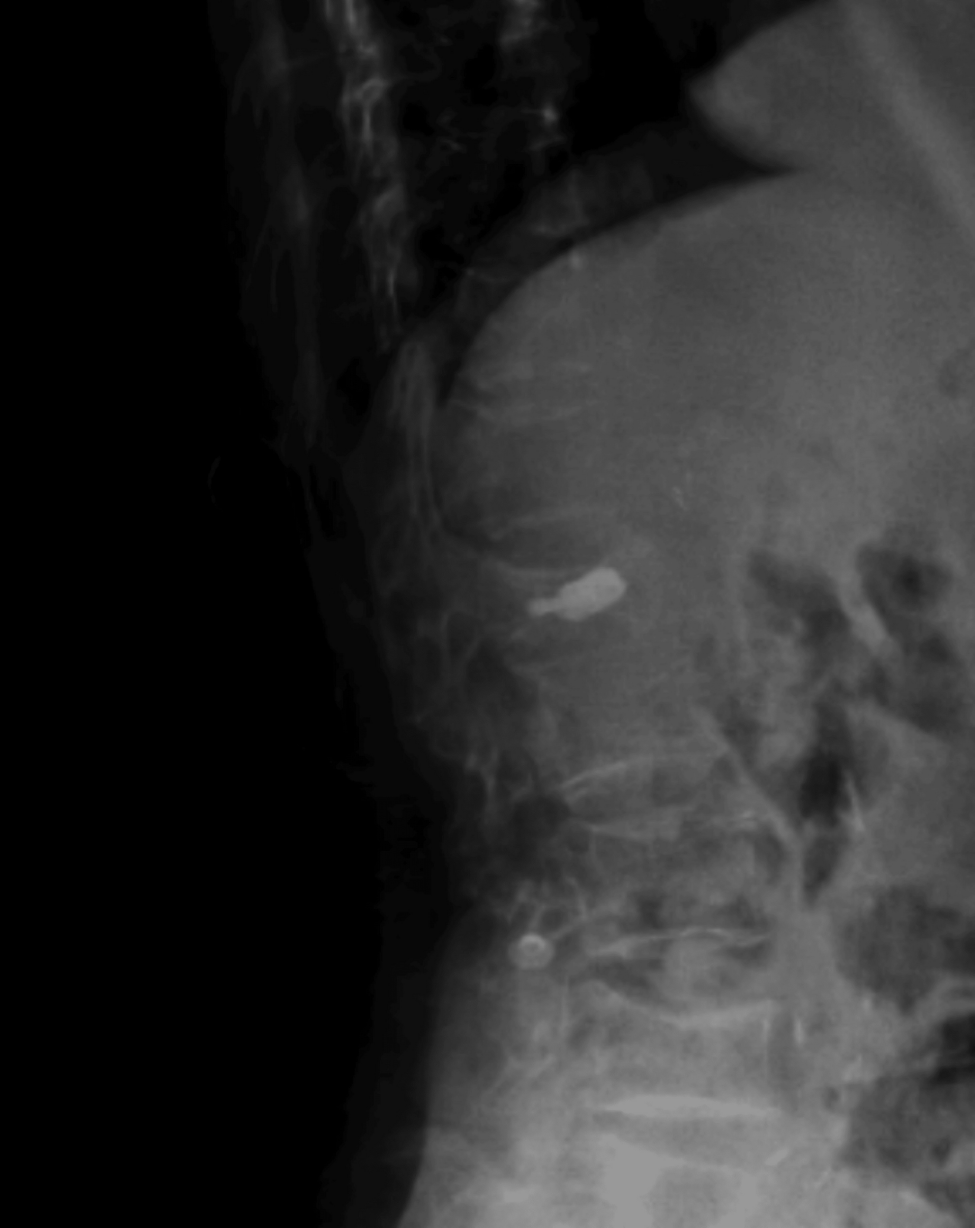
Lateral radiographs showing adequate filling of bone cement within Vessel-X in the L1 vertebral body resulting an increase in its vertical height

The patient's pain was significantly reduced after surgery. After a week, radiography revealed that L1VB height had recovered, as shown in Figure 6. The patient's follow-up appointments after one, three, and six months indicated she was healthy.

## Discussion

The thoracolumbar vertebrae of the spine are typically the site of vertebral compression fractures. These fractures are frequently the result of osteoporosis. The patient experienced constant pain at the kyphosis and fracture site, along with a loss of vertebral body height and spinal instability. These conditions greatly reduced the patient's quality of life. However, as PKP technology became more commonly used, attention was drawn to its high initial costs, the potential for weakening the reinforced vertebral body, and the increased risk of subsequent vertebral fractures. Therefore, improved techniques for managing OVCF, reducing patient pain, and improving quality of life were needed [7]. There was a significant risk of harm to the spinal cord (cauda equina) or nerves, especially if posterior wall damage was present.

The basic concept of vesselplasty was to treat kyphosis and restore the height of the compressed vertebral body by gradually expanding the distal detachable mesh container (Vessel-X). This was done by inserting the container via a working path into the injured vertebra, filling it with bone cement, and applying pressure. Once the bone cement began to cure, the container was removed, leaving the cement inside the vertebral body [8].

Studies revealed that PKP, PVP, and vesselplasty all restored vertebral body height, with vesselplasty having a reduced incidence of bone cement leakage (BCL). However, all three surgeries resulted in significant pain reduction. The Vessel-X container was considered the best option for treating spinal fractures with damage to the peripheral walls because its 100 µm mesh allowed the bone cement that leaked out to attach itself to the cancellous bone [6]. Our study found no BCL invading the spinal canal during our investigation. When spinal expansion was completed in PKP, the balloon was removed before bone cement was administered. Therefore, it was easy for the vertebra to collapse again due to the low mechanical characteristics of the void. The bone cement that was injected directly to increase pressure during vesselplasty could cause the vertebral body to expand and spread outside the container. Thus, in theory, vesselplasty could improve the restoration of vertebral body height and treat kyphosis. Further clinical trials were necessary to investigate the distinctions between vesselplasty and PKP.

Studies discovered that vesselplasty and PKP were statistically significant compared to PVP in terms of restoring vertebral body height, but the latter two did not differ. While study results varied regarding the extent of vertebral body height recovery, all indicated significant improvement above preoperative levels. Furthermore, a compressed vertebral body could result in kyphosis and a reduction in the upper and lower intervertebral discs' capacity to distribute stress, which could lead to a neighboring vertebra breaking or an injured vertebra refracturing. Thus, for the management of OVCF, the collapsed and fractured vertebral body had to be stabilized and its height had to be restored [9]. We observed a decrease in the mean Oswestry Disability Index (ODI) and Visual Analogue Score (VAS) scores of the patient implying a significant surgical improvement of the patient’s functional status along with a decrease in fracture-related pain. Based on our radiological assessment, vertebral body height was significantly enhanced, satisfactory improvement of spinal deformity was achieved, and vertebral body height was reconstructed using cement injection through a mesh container.

## Conclusions

Vesselplasty has proven to be a viable and successful treatment for VCFs, especially in patients with peripheral wall injuries who are osteoporotic. The process overcomes the drawbacks of conventional techniques like vertebroplasty and balloon kyphoplasty by improving vertebral height and total spinal stability in addition to delivering significant pain relief. In contrast to previous treatments, vesselplasty reduces the possibility of bone cement leakage by employing a polyethylene terephthalate (PET) balloon container. The results of this study demonstrate substantial improvements in pain relief, functional status, and repair of the vertebral body following surgery, indicating that vesselplasty is a good substitute for controlling VCFs. Considering the positive outcomes and lower rate of complications, vesselplasty may be a better choice for patients with VCFs, particularly those who also have osteoporosis. To firmly establish its place in the care of spinal fractures and to assess its long-term results in comparison to other proven treatments, more investigation and clinical studies are necessary.

## References

[1] Kondo KL (2008). Osteoporotic vertebral compression fractures and vertebral augmentation. Semin Intervent Radiol.

[2] Marcucci G, Brandi ML (2010). Kyphoplasty and vertebroplasty in the management of osteoporosis with subsequent vertebral compression fractures. Clin Cases Miner Bone Metab.

[3] Wang B, Zhao CP, Song LX, Zhu L (2018). Balloon kyphoplasty versus percutaneous vertebroplasty for osteoporotic vertebral compression fracture: a meta-analysis and systematic review. J Orthop Surg Res.

[4] Patel A, Petrone B, Carter KR (2024). percutaneous vertebroplasty and kyphoplasty. StatPearls [Internet].

[5] Piao M, Darwono AB, Zhu K, Zhao K (2019). Extrapendicular approach of unilateral percutaneous vesselplasty for the treatment of kummell disease. Int J Spine Surg.

[6] Xu K, Li YL, Xiao SH (2021). Vesselplasty versus vertebroplasty in the treatment of osteoporotic vertebral compression fractures with posterior wall rupture. J Int Med Res.

[7] Song Q, Zhao Y, Li D (2023). Effect of different bone cement distributions in percutaneous kyphoplasty on clinical outcomes for osteoporotic vertebral compression fractures: a retrospective study. Medicine (Baltimore).

[8] Xu C, Yin M, Mo W (2020). Vesselplasty for the treatment of osteoporotic vertebral compression fractures with peripheral wall damage: a retrospective study. Res Sq.

[9] Zhang T, Peng Y, Li J (2024). Comparison of clinical and radiological outcomes of vertebral body stenting versus percutaneous kyphoplasty for the treatment of osteoporotic vertebral compression fracture: a systematic review and meta-analysis. Jt Dis Relat Surg.

